# Phonological network fluency identifies phonological restructuring through mental search

**DOI:** 10.1038/s41598-019-52433-w

**Published:** 2019-11-05

**Authors:** Karl David Neergaard, Jin Luo, Chu-Ren Huang

**Affiliations:** 1 University of Macau, Department of English, Macau S.A.R., China; 2 Aix-Marseille University, Laboratoire Parole et Langage, Aix-en-Provence, 13100 France; 3 University of Groningen, Erasmus+ Mundus Joint Master Degree in Clinical Linguistics, 9712 Groningen, The Netherlands; 4 The Hong Kong Polytechnic University, Department of Chinese and Bilingual Studies, Hong Kong S.A.R., China

**Keywords:** Long-term memory, Human behaviour, Complex networks

## Abstract

We investigated network principles underlying mental search through a novel phonological verbal fluency task. Post exclusion, 95 native-language Mandarin speakers produced as many items that differed by a single segment or lexical tone as possible within one minute. Their verbal productions were assessed according to several novel graded fluency measures, and network science measures that accounted for the structure, cohesion and interconnectedness of lexical items. A multivariate regression analysis of our participants’ language backgrounds included their mono- or multi-lingual status, English proficiency, and fluency in other Chinese languages/dialects. Higher English proficiency predicted lower error rates and greater interconnectedness, while higher fluency in other Chinese languages/dialects revealed lower successive similarity and lower network coherence. This inverse relationship between English and other Chinese languages/dialects provides evidence of the restructuring of the phonological mental lexicon.

## Introduction

Among the stages attributed to spoken word production includes the retrieval of phonological representations followed by their transformation into an articulatory plan. According to the canonic language production models, phonological units are constructed into syllables within a metrical^1^ or phonological representational frame^2^. In European languages such as Dutch, English, and French, evidence from on-the-fly resyllabification^1^, error production^2,3^, and priming^4–7^ have supported the contention that syllables are constructed from segmental or near-segmental units. Over the last decade, however, evidence has mounted for cross-linguistic differences. Evidence from error analyses in Mandarin Chinese^8–10^, and studies implementing priming paradigms in Mandarin^11–17^, and Japanese^18,19^, showed a lack of segmental processing, motivating the claim that the first likely phonological unit for retrieval, called the ‘proximate unit’, is syllabic in Mandarin, moraic in Japanese and segmental in Dutch, English and French.

This narrative however is not without its detractors. Evidence from multiple fronts, including behavioral and neurological studies, and speech error corpora, reveal a complex story.

The premise that the proximate unit would be generalizable within the same language family did not hold for speakers of Cantonese Chinese, where segmental processing within a priming paradigm^20^ and picture-word interference tasks^21,22^ was found. This tendency towards segmentation during speech for Cantonese speakers was seen within a speech error corpus wherein errors of segments and lexical tone were the most frequent units^23^.

Mixed results have been generated from studies implementing neurological methods with Mandarin speakers. Supporting evidence for the proximate unit using electroencephalography (EEG) within a picture-name priming study^24^ stands in contrast to two EEG studies using a phoneme repetition task^25^, and a picture-name priming paradigm^26^. Meanwhile, a fourth EEG study using an interference paradigm reported unique time signatures per syllable constituents, with atonal syllables being retrieved prior to segments^27^. Similarly, a functional magnetic resonance imaging (fMRI) study using a priming paradigm^28^, found distinctive neural representations for both segments and syllables, similar to results from French^29^.

Finally, when Mandarin participants remembered lexical items as presented in either Chinese characters (syllable-sized) or pinyin (Mandarin romanization), during the form preparation paradigm, onset effects occurred when onset letters overlapped but not when onsets of Chinese characters overlapped^30^. This revealed that a principle paradigm used in the theory’s creation cues the memory of the orthographic units rather than solely their phonological representations. The connection between phonology and orthography in a recent study implementing a picture-naming form preparation task more firmly places the proximate unit within the realm of literacy acquisition. Kindergartners, who were learning via pinyin, showed onset effects, grade 1 and grade 2 students tonal syllable effects, while grade 4 students and adults, in line with the proximate unit principle, revealed atonal syllable effects^31^.

One possible explanation to account for the mixed results of the above-mentioned studies, particularly with the Mandarin speaking participants, lies in their often, rich language backgrounds. The bulk of participants tested to date have been university students with not only varying degrees of proficiency in English but other Chinese languages/dialects, which make them candidates for putative bilingual advantages or disadvantages.

Bilingual disadvantages have been attributed to lower proficiency in both languages when compared to monolinguals^32–37^, increased lexical competition^38,39^ or language attrition due to a drop in first language (L1) frequency of use during increased use of a second language (L2)^40,41^. Advantages in processing are hypothesized to be due to gains in executive control due to switching between languages^42^. Counter evidence^43–46^ has however begun to mount as to bilingualism’s putative benefits. An example of this can be seen within the verbal fluency domain where despite a number of bilingual effects in the letter fluency task, a recent meta-analysis^47^ revealed no reliable effect. The same meta-analysis however showed a bilingual disadvantage in semantic fluency.

Recent studies have also pointed to an interaction between other Chinese languages during Mandarin processing. Wu *et al*.^48^ found that bilingual speakers of both Jinan Mandarin and Mandarin (Puthonghua or Standard Chinese), which critically differ in tonal assignments per lexical items, were facilitated in lexical judgments in an auditory lexical decision task unlike their Mandarin monolingual peers. Facilitation was also seen in a speech production task wherein native-Mandarin speakers of greater proficiency in other Chinese languages were faster in producing phonological neighbors of Mandarin monosyllables^49^. These two studies support a building hypothesis that typologically similar languages facilitate rather than inhibit processing^50^.

To explain a possible influence of either English proficiency or other Chinese languages/dialects on mental search it is necessary to rely on the literature of phonological/orthographic effects. In speech recognition tasks with speakers of alphabetic scripts two proposals have taken form that entail either the cross-activation of orthographic and phonological representations^51–54^, or the restructuring of phonological representations due to the acquisition of orthography^55,56^. Mandarin has played a unique role in this literature because this population undergoes years of learning a logographic script that critically differs from learning an alphabetic script due to its demand on memory and reliance on handwriting^57^. fMRI studies have shown that phonological and orthographic brain networks activated during auditory and reading tasks across multiple age groups critically differ from those seen in English speakers^58–60^. For instance, when children and adults, each of English L1 and Mandarin L1 proficiency performed auditory rhyme judgments of orthographic/phonological inconsistency, (e.g., *pint* /pʰaɪntʰ/, *mint* /mɨntʰ/) only English adults, and children with high reading skill showed greater activation in brain areas related to phonological processing^58^. Meanwhile, in the area of speech production, there is evidence of cross-activation in picture naming^61–64^. Recent Mandarin speech production results support the implication that orthography is active during retrieval of phonological information, without providing evidence of restructuring. Naming latencies of colored line drawings were facilitated for words that shared radicals (components of Chinese characters)^64^ despite characters never being exposed to the participants during the task. Using the same paradigm, Tibetan L2 speakers of Mandarin showed that interactivity of Chinese orthography and phonological information during speech production occurs for L2 speakers as well^63^.

How might the acquisition of an alphabetic language affect Mandarin speakers? There is evidence to suggest that greater fluency leads to greater segmentation in Mandarin. Mandarin speakers of high English proficiency showed onset priming effects in Mandarin when portions of syllable structure overlapped (e.g., CV→CVN)^65^. Meanwhile, Mandarin-English bilinguals also showed priming effects in Mandarin after multiple repetitions of target items in a form preparation task^66^. A potential interpretation of these results is that greater proficiency in an alphabetic language, in which the orthography capitalizes on phonological information, leads to greater segmentation of Mandarin during the processing of Chinese characters, despite there being little transparency between Mandarin phonology and Chinese characters^67^. Rather than this interpretation being one of cross-activation, it is dependent on acquisition and subsequent proficiency in the L2, and as such represents a possible route for the restructuring hypothesis.

A current gap in the literature on phonological segmentation in speech production is the lack of investigation into the nature of the participants’ phonological representations and how such representations might vary between participants, particularly due to knowledge of other languages. One methodology being used in the study of mental representations involves the use of network science with psycholinguistic tasks^68^. In such studies lexical items are nodes and their edges a relational parameter based on linguistic aspects such as semantics^69^, phonology^70^, orthography^71^, and/or two^72^, or more layers^73,74^. The use of networks to study variation amongst a population has thus far been done through networks created from individual participants’ responses in semantic fluency tasks. Results from such studies have revealed differences between monolingual and bilingual speakers^75^, children of typical hearing and those raised with cochlear implants^76^, and healthy controls and patients of mild cognitive impairment and Alzheimer’s^77^. The innovative application of network science to semantic fluency has occurred despite the challenge of establishing which words are or aren’t neighbors (i.e., those words that share an edge)^78,79^. Contrary to this limitation, a task implementing phonological verbal fluency is uniquely fit for participant-level networks due to the modeling assumption that phonemes are mental categories shared across the speakers of a language^2,80^.

While individual networks have not yet been explored in the literature dedicated to phonological networks, a wealth of methodological and theoretical tools for the modeling of speech processing has taken shape. At the macro level, topological features have been analyzed both within^49,73,74,81,82^ and between languages^82–86^ to reveal commonalities, such as positive mixing by degree (wherein nodes with many edges tend to be neighbors of nodes with many edges). At the meso-level, analyses of communities (groups of nodes within the network)^87^, and components (subgraphs and unconnected nodes, i.e., “isolates”), suggest that words of greater connectivity are slowed in recognition and recalled less accurately^88^. At the micro level, the word-level measure known as clustering coefficient (the proportion of a node’s neighbors that are also neighbors of each other) expanded the previous understanding of interactivity between lexical items in both speech production^89^, and recognition^90,91^ by showing that a word’s interconnectedness affects lexical processing.

The use of phonological networks within a fluency paradigm builds on a long history of probing the phonological mental lexicon through the letter fluency task^92,93^. Letter fluency has participants produce words based on their sharing an alphabetic letter. Dependent variables from the task have primarily included three simple measures^94^: number of valid productions, number of clusters, and number of switches. A network phonology approach improves on the biases of the letter fluency task by making each measure quantitative and dependent on knowledge of phonology rather than orthography. For instance, in the letter fluency task, two responses are considered part of a cluster if they begin with the same two letters. This is problematic because while letters might correspond to phonemes, they often do not (e.g., cat /kʰætʰ/, car /kʰɑɹ/, cake /kʰeɪkʰ/). We replace the qualitative concept of clusters by counting our participants’ network components, and measuring their networks’ clustering coefficients (i.e., node interconnectedness). Additional measures are introduced to account for graded similarity, our participants’ switching (divergence from a cluster), and their rate of producing items with a syllable bias through measuring the consecutive production of syllable neighbors (i.e., items that share the same atonal syllable).

In the current study, we construct for the first time individual-level phonological networks wherein nodes are monosyllabic lexical items produced within a phonological verbal fluency task that asks participants to produce phonological neighbors to monosyllabic stimuli. Participants were instructed on the creation of networks’ wherein edges were defined by the relational parameter known as phonological edit distance^95^, in which two lexical items are immediate phonological neighbors (i.e., edit = 1) if they differ from one another by the addition (*at* /ætʰ/ → *cat* /kʰætʰ/), deletion (*cat* /kʰætʰ/ → *at* /ætʰ/), or substitution (*mat* /mætʰ/ → *cat* /kʰætʰ/) of a single phonological segment or lexical tone (*ma1*/ma^55^/ → *ma3*/ma^214^/).

We hypothesized that by giving instructions to produce neighbors that required the segmentation of the target stimuli we would induce differential results between those participants that tend toward segmentation and those that tend toward syllable processing. We first described our participants’ networks through a correlation analysis and visualization of example networks in order to identify whether participants used a search strategy indicative of mental search of a segmental or syllabic nature. In terms of network structure, we predicted our participants’ networks would vary in clustering coefficient, number of network components and particularly mixing by degree, where we assumed networks would reveal both positive (assortative) and negative (disassortative) values contrary to the results reported from whole-vocabulary phonological networks. We then performed a multivariate analysis to investigate whether our participants’ language backgrounds influenced search through the mental lexicon. Because of the rather mixed results in regards to bilingualism, and specifically the null effect in letter fluency, we were agnostic as to its possible effects. However, we hypothesized that differential effects for fluency and network measures due to both English proficiency and knowledge of other Chinese languages/dialects would present a case for phonological restructuring. We hypothesized that speakers with greater fluency in other Chinese languages/dialects would be biased towards mental search of syllable sized units and thus tend toward a greater proportion of syllable neighbors. A greater proportion of syllable neighbors would in turn result in low clustering coefficient, a high number of network components, a higher mean rate of switching, and greater errors, seeing as many items within a syllable family are nonitems. Meanwhile, we predicted that participants with higher English proficiency would tend towards greater segmentation (i.e., low edit distance between target stimuli and verbal productions), which would lead to high clustering coefficient, a lower number of network components and fewer errors due to their greater facility in segmenting related to proficiency in processing an alphabetic language.

## Methods

### Participants

Post-exclusion, 95 adult Mandarin speakers (Female: 66; Ages 18–38, M: 23.44; SD: 4.16), recruited from the Hong Kong metropolitan area, were included in this study. Before beginning the experiment, participants completed a short biographical survey which included, besides age and sex, the name of their home province, self-rated spoken proficiency on a scale of 1 (beginner) to 10 (native speaker) in English (levels 5–8, M: 6.74; SD: 0.94) and other Chinese languages/dialects and/or other non-Chinese languages.

Self-assessed English proficiency was used in the current study. Given the inordinate cost and duration of administration of language proficiency instruments (e.g., 2–5 hours at $799^96^), self-assessment is used as a replacement^97,98^, particularly in low-stakes and low-resource settings. Despite their limitations, self-assessment has shown reliability and validity as a measure of oral proficiency in regards to other direct methods^99–101^.

All participants reported native-level proficiency in Mandarin with no history of speech or hearing disorders. To create a rough representation of knowledge of other Chinese languages, we summed the number of Chinese languages/dialects (Num_Chinese) that fell within the self-rated values of 3–10 (levels 1–3; M: 1.92; SD: 0.66). Num_Chinese was recently found to significantly facilitate the spoken production of phonological neighbors^49^. From our participants’ spoken language proficiency ratings, we created the variable, Multilingual, through categorizing speakers as either monolingual (48), for ranking only one language between 9–10, or multilingual (47), for ranking more than one language between 9–10. Note that due to only five participants ranking three languages between 9–10 in fluency, we did not create bilingual and trilingual categories, but collapsed them into the single multilingual category.

From the original 107 participants recruited, one participant was excluded due to researcher error in acquiring demographic data. Seven participants were excluded due to excessive error rates lying 2.5 standard deviations above the group mean for productions of nonwords (5) and repetitions (2). A further four participants were excluded due to being the sole participants in English proficiency levels 1, 4 and 9, and Num_Chinese level 4.

The Hong Kong Polytechnic University’s Human Subjects Ethics Sub-committee (reference number: HSEARS20140908002) reviewed and approved the details pertinent to all experiments conducted in this study prior to beginning recruitment. The methods were carried out in accordance with guidelines and regulations. The participants gave their informed consent and were compensated with 50HKD for their participation.

### Stimuli

The lexical statistics to describe our stimuli and later our participants’ word-level networks come from the tonal fully segmented schematic representation (C_G_V_X_T) found in the updated version of the Database of Mandarin Neighborhood Statistics^102^. The current instantiation of the database is freely available here: https://github.com/karlneergaard/Database_of_word-level_statistics.

The stimuli consisted of 6 monosyllabic Mandarin words that were high in single edit neighbors (M: 21.67; SD: 4.13), and differed in syllable structure: *ye1* (/iɛ^55^/, GV, edit 1 = 18); *an3* (/an^214^/, VX, edit 1 = 23); *wai4* (/uaɪ^51^/, GVX, edit 1 = 29); *du2* (/tu^35^/, CV, edit 1 = 20); *guo4* (/kuo^51^/, CGV, edit 1 = 22); *ceng1* (/ tsʰəŋ^55^/, CVX, edit 1 = 18). Their variation in syllable structure, while holding edit high, was done in order to eliminate overlap in hop 1 and hop 2 neighbors.

### Procedure

Seated in a quiet room and wearing headphones equipped with an adjustable microphone, each participant was exposed to 8 short videos featuring a male Mandarin speaker in his 20s from the Beijing area that verbally provided the experiment’s instructions, practice, and six stimuli. During the experiment’s instructions phase, participants were told to produce as many phonological neighbors to a given stimuli as possible within 1 minute. To illustrate what qualified as phonological neighbors the speaker presented the monosyllable *jie1* /tɕiɛ^55^/ and neighbor examples according to the replacement of the glide, *jue1* /tɕyɛ^55^/, the addition of a final nasal, *jian1* /tɕiɛn^55^/, the replacement of the onset, *xie1* /ɕiɛ^55^/, replacement of the monophthong, *jia1* /tɕia^55^/, and the substitution of lexical tone, *jie2* /tɕiɛ^35^/. The practice phase had participants produce phonological neighbors to the monosyllable *jing3* /tɕiŋ^214^/ for 1 minute. Prior to beginning the experiment participants were instructed to not produce non-items, which included syllables that do not correspond to existing Chinese characters. Participants had one minute for each of the six randomized stimuli.

### Measures

**Figure 1:**
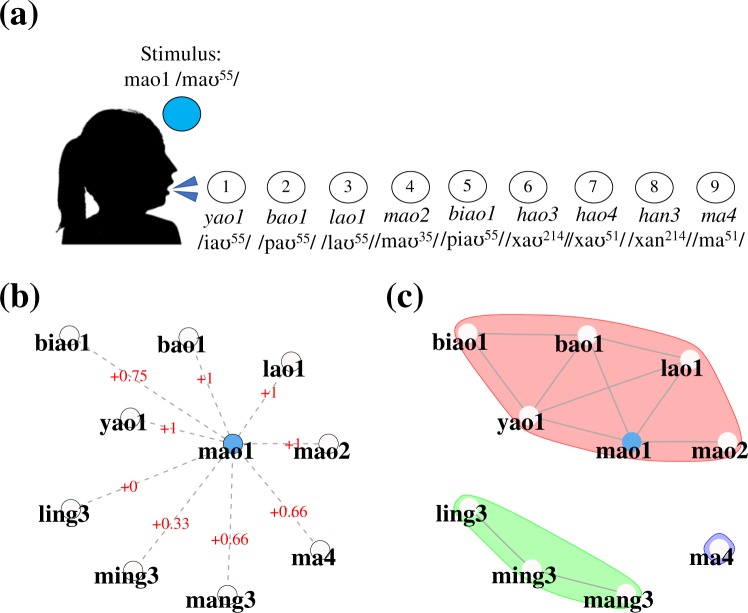
(**a**) Example stimulus and responses within the phonological network fluency task, according to both (**b**) the weighted edit (WE) metric, and (**c**) the number of components (*NC*) within a given network.

The current measures depend on shared phonological similarity between participants’ verbal productions and an experimenter-provided stimulus, as is illustrated in Fig. 1a.

#### Error

Verbal fluency tasks require participants to simultaneously suppress and retain lexical items in working memory, which draws from executive control processes^103^. To assess the level of executive control used during the task we summed both error responses (Error): repetitions and nonword productions.

#### Fluency

We introduce ‘weighted edit’ (WE), shown in Fig. 1b. WE states that a given word’s value to the final fluency score decreases as edit distance between itself and the auditory stimuli increases. Fluency weightings differed according to the number of units for each of the stimuli and production, such that weightings were the proportion of the number of edit distances possible prior to two items sharing no similarity. For instance, for four-unit stimuli that received five-unit responses, edit 1 garnered 1 point (e.g., wai4 /uaɪ^51^/→ shuai4 /ʂuaɪ^51^/), edit 2 = 0.75 (e.g., wai4 /uaɪ^51^/→ shuan4 /ʂuan^51^/), edit 3 = 0.50 (e.g., wai4 /uaɪ^51^/→ xiang4 /ɕiaŋ^51^/), edit 4 = 0.25 (e.g., wai4 /uaɪ^51^/→ xiang3 /ɕiaŋ^214^/), and edit 5 = 0 (e.g., wai4 /uaɪ^51^/→ xiong3 /ɕioŋ^214^/). Similarly, for three-unit stimuli that received two-unit responses, edit 1 = 1 (e.g., du2 /tu^35^/ → wu2 /u^35^/), edit 2 = 0.50 (e.g., tu2 /tu^35^/ → wu4 /u^51^/), edit 3 = 0 (e.g., tu2 /tu^35^/ → yi4 /i^51^/), edits 4 and 5 also received 0 points due to not being possible combinations.

In order to capture similarity over time, and thus a participant’s rate of switching, we introduce the measure, ‘running edit’ (RE). RE entails the mean of successive weighted edit distances of all items produced per trial. Similar to the method of measuring switching^94^, nonword productions and repetitions were included in its calculation. Note that an RE of 1 means that every successive production was an immediate neighbor of the one that preceded it, while lower values mean that mental jumps were made that repetitively deviated from successive similarity.

Given the question of whole-syllable retrieval specific to Mandarin speakers, and the known tendency to manipulate lexical tone while maintaining the syllable in phonological association tasks^49,104^, we devised a variable that would test if successive productions were syllable neighbors (SN). SN entails the proportion of successive syllable neighbors within a trial including repetitions and nonwords.

#### Network

Correct lexical items per each trial were constructed into undirected graphs wherein two given items shared an edge if they had an edit distance of one. Mean clustering coefficient ($\overline{CC}$), mixing by degree (*M*), and the number of components per network (*NC*) were calculated through the use of the igraph package in R^105^.

At the word level, clustering coefficient (*CC*), is the proportion of neighbors who are also neighbors of each other and represented as falling between 0 and 1. In studies that contrasted the effects of high versus low *CC*, words high in *CC* have been tied to greater speech errors^89^, lower accuracy in auditory perception^90^ and lexical judgment^90,106^, slower reaction times in picture naming^89^, yet greater consolidation of novel words^107^. At the network level, $\overline{CC}$ is a global measure of interconnectivity between nodes calculated by averaging local *CC* across all nodes of the giant component. Phonological networks, representing a number of languages, have shown $\overline{CC}$ values of between 0.191 and 0.628^73,74,81–84,86^, including Mandarin^49^.

A network’s *M* is represented as falling between -1 and 1. Negative *M* values are referred to as disassortative. They occur when networks have star like patterns, i.e, wherein few nodes are connected to many nodes. Positive *M* values are referred to as assortative. They occur when high degree nodes are connected to other high degree nodes. We included this measure in the current study because any deviation from assortativity, which has been found in all phonological networks investigated to date^73,74,81–84,86^, including Mandarin^49^, would be indicative of differences between participant-level and whole vocabulary investigations of the phonological mental lexicon.

Knowing from previous association tasks^49,104^ that Mandarin speakers produce edit distances greater than one when asked to produce minimal pairs, we assumed our participants’ networks would feature both disconnected lexical items (isolates) or separate groupings of items (islands). Our final dependent variable, number of components (*NC*), is a quantitative account of the number of clusters per verbal productions. It represents the success (few components) or failure (many components) to produce a coherent network. An example network with an *NC* of three can be seen in Fig. 1c.

The custom in the analysis of networks is to exclude isolates/islands and then report values for the largest connected component. This tradition is due to calculations diverging to infinity without an available edge between components. In order to represent all given correct lexical items, while simultaneously penalizing poorer performance marked by the presence of islands/isolates, we calculated a weighted average across all components for both $\overline{CC}$ and *M*, wherein isolates were set to zero. Thus, if in a 10-node network, wherein *component A* has a $\overline{CC}$ of 1 with 9 nodes, and *component B* is an isolate, the weighted $\overline{CC}$ value would be reduced by 10% and equal 0.9.

## Results

Responses were transcribed to pinyin by two native-Mandarin speaking volunteers. Using the above-mentioned database^102^, items were categorized as either real lexical items, or nonwords. Real lexical items consisted of syllables that could be ascribed to at least one Chinese character whether or not they qualified as monosyllabic words. Items were classified as correct responses if they did not correspond to a nonword in the database or were not repetitions.

Exclusions of outliers were made according to trials that lied 2.5 standard deviations above (WE: 11 trials; SN: 1 trial) and below (RE: 12 trials) the mean, and through the use of a boxplot: *M* (4 trials). Total number of exclusions (28) accounted for 4.9% of all trials. According to the same criteria, no exclusions were made based on the distributional properties of $\overline{CC}$. Finally, no exclusions were made based on *NC* or Error, because their distributional features were addressed in the statistical models to follow. Table 1 displays both correlations between the dependent variables and their descriptive attributes by min, max, mean, and standard deviation.

**Table 1:** Summary of correlations and distributional information of fluency, error, and network variables.

	Correlations						Descriptives	
	WE	RE	SN	Error	$\overline{CC}$	*M*	Min:Max	M (SD)
WE							1:18.33	8.73 (3.35)
RE	0.39						0.50:1	0.80 (0.11)
SN	0.35	0.40					0:0.80	0.37 (0.25)
Error	0.15	0.10	0.26				0:16	1.80 (2.11)
$\overline{CC}$	0.24	0.51	−0.16	−0.09			0:1	0.49 (0.23)
*M*	0.58	0.24	0.22	0.11	0.19		−0.71:0.66	−0.02 (0.27)
*NC*	−0.28	−0.63	−0.37	−0.05	−0.33	−0.16	1:10	2.12 (1.39)

**Figure 2:**
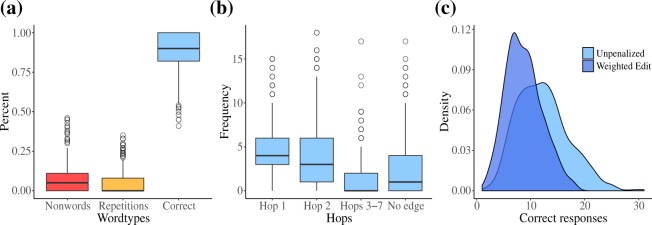
(**a**) Proportions of word types produced. (**b**) Frequency of occurrence of correct responses as measured by their hops from the target stimuli. (**c**) Density of correct unpenalized responses and weighted edit (WE).

As is shown in Fig. 2a, nonwords and repetitions were proportionally rarer than correct responses. Meanwhile, in Fig. 2b we see that participants by and large produced phonologically similar responses, such that hop-1 responses (40%, M: 4.87; SD: 2.70) were more numerous than those of hop-2 responses (31%, M: 3.83; SD: 3.65). Figure 2b also reveals that while responses were up to seven hops in distance from the stimuli, nodes that did not share an edge with the stimuli were more common per trial (19%, M: 2.34; SD: 3.26). Figure 2c illustrates the distributional difference between WE and unpenalized correct responses. As a result of penalizing neighbors with greater edit distances, WE has a lower mean and shows greater kurtosis than unpenalized correct responses (M: 12.24; SD: 4.72).

### Search strategies

Patterns within the data suggested diverging search strategies. While the positive correlation between fluency and hop-1 neighbors (WE & Hop 1 = 0.67) suggests that immediate neighbors were the target for many high fluency networks, the equally high correlation between hop-2 neighbors (WE & Hop 2 = 0.68) suggests that some participants tended toward a pattern that eschewed immediate neighbors. Evidence of a specific search strategy is clear when considering the difference in correlation between hop-1 and hop-2 responses and syllable neighbor production (SN & Hop 1 = 0.03; SN & Hop 2 = 0.57). While a syllable-driven search method is evident in the data, it was not the dominant search method. A split of all trials based on SN > 0.50, revealed that 34% of trials were created with a predominantly syllable-driven search method.

**Figure 3:**
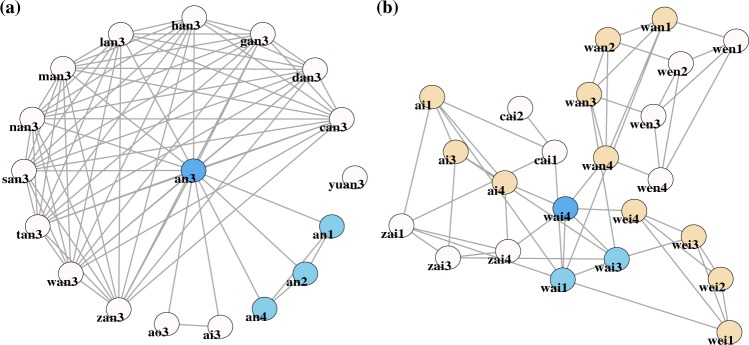
Example high-fluency networks that either (**a**) exploited knowledge of syllable constituents or (**b**) exploited knowledge of the Mandarin syllable inventory, as noted by groups of syllable neighbors. Color coding was used to denote the stimuli (dark blue), the stimuli’s syllable neighbors (light blue), and whether the participant produced multiple items from syllable families (alternating white and light brown).

In Fig. 3 we illustrate two search methods that produced networks of equivalent fluency scores (3a, WE = 15.66; 3b, WE = 15.57), despite not having an equivalent number of correct responses (3a = 16; 3b = 22). In Fig. 3a, we see that the participant utilized their awareness of syllable constituents in their maintenance of the rime *an3* /an^214^/ while primarily manipulating onsets. This led to a network high in interconnectedness ($\overline{CC}$ = 0.914), with a low proportion of syllable neighbors (SN = 0.176), consisting of fifteen hop-1 responses and zero hop-2 responses. In Fig. 2b we see an example of a syllable-driven search, wherein the identification of an immediate neighbor (e.g., *wai4* /uaɪ^51^/ → *zai4* /tsaɪ^51^/) brought with it syllable neighbors (*zai1, zai2, zai3*) whether or not they were non-items (*zai2*). In contrast to the network in Fig. 3a, the syllable driven method seen in 3b resulted in lower $\overline{CC}$ (0.391) and a much higher proportion of syllable neighbors (SN = 0.760), consisting of seven hop-1 responses, twelve hop-2 responses, and four hop-4 responses.

To understand the roles of RE, and *M* it is necessary to look at the graphs in terms of the correlation analysis. As is evident from their respective RE values (3a, RE = 0.87; 3b, RE = 0.89), both search methods can produce high RE values. From the correlations in Table 1 we see that rather than inform us on the use of syllables as a means of search, RE entails search methods that either closely tie to phonological similarity (low RE) and thus result in high $\overline{CC}$, or deviate from successive similarity (high RE) and result in less network coherence (high *NC*). Meanwhile, the graphs’ values for *M* (3a, *M* = 0.37; 3b, *M* = 0.23) support the positive correlation between greater fluency (high WE) and positive *M* values, illustrating that the likelihood of assortativity increases with a greater number of responses.

### Statistical analysis

The statistical analysis utilized the mcglm package in R^108^, which allowed for the fitting of regression models with multiple dependent variables, and distributional family types. Of the fluency (WE, RE, SN), and network variables ($\overline{CC}$, *NC*), only *M* was best fit with a normal distribution. The tweedie variance function^109^ was used to fit WE, RE, SN and $\overline{CC}$, while the poisson-tweedie variance function^110^ was used to fit the count variables, *NC* and Error.

As can be seen in Table 2, higher self-rated spoken-English proficiency showed a facilitative effect on greater $\overline{CC}$. The proportion of syllable neighbors and errors produced by participants also significantly predicted English proficiency, such that higher proficiency participants produced less syllable neighbors (lower SN), and fewer errors than lower proficiency speakers. Greater Num_Chinese equated both the production of less successively similar items (RE), and less successive syllable neighbors (SN). Networks of less coherence (high *NC*) were also produced by participants with greater Num_Chinese. Finally, multilingual speakers on average produced less errors (Multilingual, M: 1.66; SD: 1.85; Monolingual, M: 1.93; SD: 2.33). No effects were found for either WE, or *M*.

**Table 2:** Effects of language background on phonological fluency.

	English				Num_Chinese				Multilingual			
	Chi	Est.	t	p	Chi	Est.	t	p	Chi	Est.	t	p
WE	0.60	−1.3^e-2^	−0.77	0.440	2.05	4.2^e-2^	1.43	0.152	3.39	−7.2^e-2^	−1.84	0.066
RE	2.06	8.5^e-3^	1.44	0.151	6.12	−2.5^e-2^	−2.47	**0.013**	1.05	1.4^e-2^	1.02	0.306
SN	16.84	−1.2^e-1^	−4.10	**<0.001**	6.41	−1.3^e-1^	−2.53	**0.011**	0.11	−2.3^e-2^	−0.33	0.741
Error	8.97	−1.5^e-1^	−3.00	**0.003**	1.92	1.2^e-1^	1.38	0.166	4.69	2.5^e-1^	−2.17	**0.030**
$\overline{CC}$	23.59	1.0^e-1^	4.86	**<0.001**	0.44	−2.3^e-2^	−0.66	0.507	0.62	3.8^e-2^	0.79	0.430
*M*	0.42	−8.0^e-3^	−0.65	0.517	0.38	1.3^e-2^	0.61	0.540	6.0^e-4^	7.0^e-4^	0.03	0.980
*NC*	0.39	−1.8^e-2^	−0.62	0.532	7.20	1.3^e-1^	2.68	**0.007**	2.13	−9.5^e-2^	−1.46	0.145

**Figure 4:**
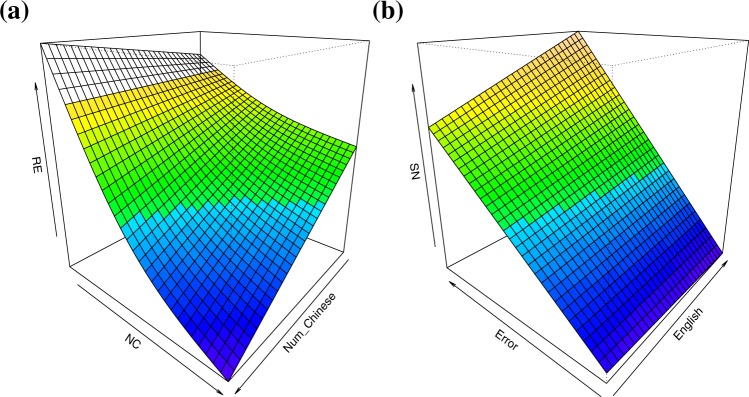
Tensor product smooths of three-way interactions between: (**a**) the effect of the number of Chinese languages spoken (Num_Chinese) and number of components produced (*NC*) on running edit (RE); (**b**) the effect of Error and English on the production of syllable neighbors produced (SN). Note that the background blends from cool to warm colors, signifying a shift from low (blue) to high values (orange) of each featured dependent variable (RE and SN).

To disentangle the effects displayed in Table 2, in Fig. 4 we graphed interactions with tensor product smooths through the use of generalized additive models within the mgcv package in R^111^.

Figure 4a illustrates that higher Num_Chinese participants produced low successive similarity (low RE), and a greater proportion of network components (high *NC*), while simultaneously illustrating the strong negative correlation between *NC* and RE previously shown in the correlation analysis. Contrary to our predictions, high Num_Chinese did not equate a tendency toward the syllable-driven search method. This means, that high Num_Chinese participants predominantly choose items that were neither syllable neighbors nor immediate neighbors, resulting in low network coherence (high *NC*).

In contrast to Num_Chinese, in Figure Fb we see that greater proficiency in English resulted in networks of greater precision. While this is implied in the significant $\overline{CC}$ effect, it is also evident in an interaction between SN, Error, and English. Figure 4b shows that higher proficiency English speakers produced less syllable neighbors (low SN), and that as SN increased so did the proportion of errors.

## Discussion

In the current study we asked if variation in phonological representations of Mandarin-speaking participants, elicited through a novel verbal fluency task, would inform on the question of segmentation in speech production. Mixed results in speech production studies, and evidence of segmentation among both speakers of high English fluency^65^, and young participants learning through pinyin^31^, led us to hypothesize that differential results would arise due to biases towards either segmental or syllable-driven search through the mental lexicon. We further hypothesized that these search methods would be due to the influence of our participants’ language backgrounds, namely, their self-reported English proficiency (English), number of other Chinese languages/dialects spoken (Num_Chinese), and the number of languages spoken with native-level proficiency (Multilingual). Our analysis revealed variation in network structure, distinct mental search methods, an effect of error for multilingual speakers, and an almost inverse relation between English and Num_Chinese.

Due to the novelty of implementing participant-level phonological networks, we begin by discussing the network measures. As expected, both mean clustering coefficient ($\overline{CC})$ and number of network components (*NC*) varied across participants. Of methodological and theoretical import are the findings related to mixing by degree (*M*). All previous measures of *M* with whole-vocabulary phonological networks have shown positive mixing by degree^49,73,74,81–84,86^, also known as assortativity. Assortativity describes the state in which nodes of high degree tend to be connected to other high degree nodes while disassortativity entails high degree nodes connected to many low degree nodes^112^. Vitevitch^81^ theorized that in an assortative network the spread of activation during lexical selection would be restricted due to high clustering, effectively lessening the number of lexical candidates, while in a disassortative network, activation would be spread throughout the mental lexicon leading to the need to reject a greater number of lexical candidates. Vitevitch and colleagues^113^ later found evidence of assortativity through investigating failed lexical retrieval through simulations with jTRACE^114^ and a number of psycholinguistic tasks that used phonological degree values extracted from the 1967 version of *Webster’s Seventh Collegiate Dictionary*.

In contrast to past investigations on phonological networks, our participants’ networks were of equal assortative and disassortative proportions. On the one hand, this difference between whole-vocabulary measures and the current variation in *M* highlights a need to account for individual differences in cognitive modeling^115^. Variation in structure according to *M* would imply speakers experience differential lexical competition and facilitation during selection due to how information spreads across lexical networks. This has been implied in cross-linguistic differences in the effect of phonological degree (i.e., phonological neighborhood density) where high degree has been shown to slow recognition in English^95,116^, but speed recognition in both Mandarin^117^ and Spanish^118^. On the other hand, the positive correlation seen between fluency (weighted edit: WE) and *M* suggests that disassortativity might be a result of low fluency rather than the structure of the lexicon. Future work can resolve the second implication simply through manipulating task duration, while the first implication will need to be addressed through investigating a greater number of individual differences than were addressed in the current study.

Through graph visualization and correlation analysis we identified two distinct patterns of search: one in which syllable constituents such as onsets, tones, and rimes were manipulated to create immediate neighbors, and the other in which all four tones of a given atonal syllable were successively produced. Both the segment- and syllable-driven search methods were successful at producing high-fluency networks. Contrary to our expectations, the successive production of syllable neighbors (SN) correlated with a high rate of successive similarity (running edit: RE), and a low number of network components. However, in line with our predictions was that this search method resulted in more errors and low clustering coefficient. A split of the distribution based on SN > 0.50 revealed that the syllable-driven method accounted for roughly 34% of all trials. In light of the proximate unit principle^14,15^, it would appear that while there was a clear influence of atonal syllables on search, it was less influential than our participants’ ability to segment. However, one limitation important to consider is that given the task instructions purposefully biased participants toward segmentation we do not know whether an opposite bias towards syllable neighbors would lead to a lower proportion of immediate neighbors. Future research would benefit from investigating whether participant’s fluency and network structure alter if given a task that involves the manipulation of syllable targets rather than segments.

The current evidence shows that variation exists among Mandarin speakers in the segmentation of syllabic constituents during speech production. Our investigation into our participants’ language backgrounds sheds light on how that variation might have taken form. While the findings of English and Num_Chinese are note-worthy, those of Multilingualism are lacking. Of the seven dependent variables analyzed only error showed a slight multilingual advantage. On the one hand this could be used to argue for a benefit of executive function. Yet, given the null effect of letter fluency in the most recent meta-analysis^47^, and the growing evidence that bilingual/multilingual effects are best explained by alternative hypotheses, such as social demographics^45,46^, intelligence^45^, or culture^44^, a multilingual advantage in verbal fluency, according to a notably small effect, should be viewed skeptically. The effects of English proficiency and Num_Chinese, however, present a case for phonological restructuring seen through network precision.

Greater knowledge of other Chinese languages/dialects led to networks of less precision. Higher Num_Chinese led to lower successive similarity between responses, and lower network cohesion (high *NC*). Surprisingly, greater knowledge of other Chinese languages also equated a lower proportion of successive syllable neighbors. In short, participants from the three levels of Num_Chinese predominantly chose items that were neither predominantly syllable neighbors nor immediate neighbors. Meanwhile, low network coherence for high Num_Chinese speakers points towards greater competition between lexical items from other Chinese languages/dialects during the task. This stands in contrast to the accounts of facilitation in lexical selection seen in previous studies^48,49^. However, it should be noted that those tasks differed from the current fluency paradigm and reflected facilitation through speed of reaction time.

The influence of greater English proficiency on our participants’ networks is one of greater precision. Higher proficiency English speakers produced a lower proportion of successive syllable neighbors, fewer errors, and more interconnected networks than lower-proficiency speakers. What can be gleaned from this knowledge is that lower-proficiency speakers drew on less knowledge of manipulating phonemic information due to less experience with an alphabetic language. Thus, rather than depending on segmentation of phonological units that in turn create highly interconnected networks, our lower-proficiency participants tended toward the use of atonal syllables and thus the increased likelihood of producing errors that comes with the syllable-driven search method.

While the contrast in network precision between English and Num_Chinese is evidence of the orthographic restructuring of the phonological mental lexicon, there are limitations to be considered. The first lies in our use of self-reported English proficiency, which does not account for the multi-dimensionality of assessing language proficiency^119^. Future research might focus on particular aspects of proficiency where segmentation is expected to affect learning outcomes. Next, due to the task design, we were unable to account for why participants of greater Num_Chinese experienced greater lexical competition. For instance it is possible that participants consciously or unconsciously used knowledge of radicals during search^63,64^. Given the constraints of the task, and the low phonological consistency of radicals, this search criteria would likely lead to spurious neighbors and thus the effect of producing neither high successive neighbors nor immediate neighbors. This is however speculation seeing as the current design was not able to account for the orthographic nature of our participants’ responses in light of any given spoken response corresponding to numerous homophones.

In summary, the current study expanded on the explanatory capabilities of a phonological fluency task through the use of network science and phonological edit distance. The edit distance metric allowed for a graded assessment of fluency but more importantly, quantifiable means to assess the contents and structure of our participants’ responses. Critical to the evidence to support the claim of phonological restructuring, was the ability to quantify successive similarity, network coherence, and node interconnectedness. It is prudent to point out that the identification of restructuring is only likely to occur with language pairs such as English and Mandarin, specifically due to the contrast in orthographic systems. Future uses of phonological network fluency would benefit from applications to both languages other than Mandarin and to clinical populations wherein graded fluency and network characteristics can inform on the knowledge and access of phonology.

## Data Availability

The data pertaining to the current study can be accessed in both the raw responses per participant, and the processed data per trial here: https://github.com/karlneergaard/phonological_network_fluency.

## References

[1] Levelt, W. J. M., Roelofs, A. & Meyer, A. S. A theory of lexical access in speech production. *Behav. Brain Sci*. **22**, 1–38, discussion 38-75 (1999). 10.1017/s0140525x99001776 11301520

[2] Dell GS (1986). A spreading-activation theory of retrieval in sentence production. Psychol. Rev..

[3] Shattuck-Hufnagel, S. Speech errors as evidence for a serial-ordering mechanism in sentence production. in Sentence processing: Psycholinguistic studies presented to Merrill Garrett (eds Cooper, W. E. & Walker, E. C. T.) 295–342 (Erlbaum, 1979).

[4] Meyer AS, Schriefers H (1991). Phonological facilitation in picture-word interference experiments: Effects of stimulus onset asynchrony and types of interfering stimuli. J. Exp. Psychol. Learn. Mem. Cogn..

[5] Schiller NO (2000). Single Word Production in English: The Role of Subsyllabic Units During Phonological Encoding. J. Exp. Psychol. Learn. Mem. Cogn..

[6] Schiller NO (2004). The onset effect in word naming. J. Mem. Lang..

[7] Alario FX, Perre L, Castel C, Ziegler JC (2007). The role of orthography in speech production revisited. Cognition.

[8] Chen J-Y (1993). A small corpus of speech errors in Mandarin Chinese and their classification. Word Chinese Lang..

[9] Chen J-Y (1999). The representation and processing of tone in Mandarin Chinese: Evidence from slips of the tongue. Appl. Psycholinguist..

[10] Chen J-Y (2000). Syllable errors from naturalistic slips of the tongue in Mandarin Chinese. Psychologia.

[11] Chen J-Y, Lin W-C, Ferrand L (2003). Masked Priming of the Syllable in Mandarin Chinese Speech Production. Chinese. J. Psychol..

[12] Chen J-Y, Chen T-M, Dell GS (2002). Word-Form Encoding in Mandarin Chinese as Assessed by the Implicit Priming Task. J. Mem. Lang..

[13] Chen T-M, Chen J-Y (2013). The syllable as the proximate unit in Mandarin Chinese word production: An intrinsic or accidental property of the production system?. Psychon. Bull. Rev..

[14] O’Séaghdha, P. G. & Chen, J.-Y. Toward a Language-General Account of Word Production: The Proximate Units Principle. In CogSci… *Annual Conference of the Cognitive Science Society* July-Augus, 68–73 (Cognitive Science Society, 2009). PMC4701055 26744737

[15] O’Séaghdha PG, Chen J-Y, Chen T (2010). Proximate Units in Word Production: Phonological Encoding Begins with Syllables in Mandarin Chinese but with Segments in English. Cognition.

[16] You, W., Zhang, Q. & Verdonschot, R. G. Masked Syllable Priming Effects in Word and Picture Naming in Chinese. *PLoS One* **7** (2012). 10.1371/journal.pone.0046595 PMC3466322 23056360

[17] Zhang Qingfang, Damian Markus F. (2019). Syllables constitute proximate units for Mandarin speakers: Electrophysiological evidence from a masked priming task. Psychophysiology.

[18] Kureta Y, Fushimi T, Tatsumi IF (2006). The functional unit in phonological encoding: Evidence for moraic representation in native Japanese speakers. J. Exp. Psychol. Learn. Mem. Cogn..

[19] Verdonschot RG (2011). The functional unit of Japanese word naming: Evidence from masked priming. J. Exp. Psychol. Learn. Mem. Cogn..

[20] Wong AW-K, Huang J, Chen H-C (2012). Phonological Units in Spoken Word Production: Insights from Cantonese. PLoS One.

[21] Wong AW-K, Chen H-C (2008). Processing segmental and prosodic information in Cantonese word production. J. Exp. Psychol. Learn. Mem. Cogn..

[22] Wong AW-K, Chen H-C (2009). What are effective phonological units in Cantonese spoken word planning?. Psychon. Bull. Rev..

[23] Alderete, J., Chan, Q. & Yeung, H. H. Tone slips in Cantonese: Evidence for early phonological encoding. *Cognition* **191** (2019). 10.1016/j.cognition.2019.04.021 31302321

[24] Wang J, Wong AW, Wang S, Chen H (2017). Primary phonological planning units in spoken word production are language-specific: Evidence from an ERP study. Sci. Rep..

[25] Qu Q, Damian MF, Kazanina N (2012). Sound-sized segments are significant for Mandarin speakers. Proc. Natl. Acad. Sci. USA.

[26] Yu M, Mo C, Mo L (2014). The Role of Phoneme in Mandarin Chinese Production: Evidence from ERPs. PLoS One.

[27] Feng C, Yue Y, Zhang Q (2019). Syllables are Retrieved before Segments in the Spoken Production of Mandarin Chinese: An ERP Study. Sci. Rep..

[28] Yu M, Mo C, Li Y, Mo L (2015). Distinct Representations of Syllables and Phonemes in Chinese production: Evidence from fMRI adaptation. Neuropsychologia.

[29] Peeva MG (2011). Distinct representations of phonemes, syllables, and supra-syllabic sequences in the speech production network Maya. Neuroimage.

[30] Li C, Wang M, Idsardi W (2015). The effect of orthographic form-cuing on the phonological preparation unit in spoken word production. Mem. Cognit..

[31] Li C, Wang M (2017). The influence of orthographic experience on the development of phonological preparation in spoken word production. Mem. Cognit..

[32] Bialystok E, Craik FIM, Luk G (2008). Lexical access in bilinguals: Effects of vocabulary size and executive control. J. Neurolinguistics.

[33] Rosselli M (2000). Verbal Fluency and Repetition Skills in Healthy Older Spanish – English Bilinguals. Appl. Neuropsychol..

[34] Rosselli M (2002). A cross-linguistic comparison of verbal fluency tests. Int. J. Neurosci..

[35] Luo L, Luk G, Bialystok E (2010). Effect of language proficiency and executive control on verbal fluency performance in bilinguals. Cognition.

[36] Portocarrero JS, Burright RG, Donovick PJ (2007). Vocabulary and verbal fluency of bilingual and monolingual college students. Arch. Clin. Neuropsychol..

[37] Gollan TH, Montoya RI, Werner GA (2002). Semantic and Letter Fluency in Spanish – English Bilinguals. Neuropsychology.

[38] Kroll, J. F. & Gollan, T. H. Speech planning in two languages: What bilinguals tell us about language production. in The Oxford handbook of language production (eds Kroll, J. F., Gollan, T. H., Goldrick, M., Ferreira, V. & Miozzo, M.) 165–181 (Oxford Library of Psychology, 2014).

[39] Green DW (1998). Mental control of the bilingual lexico-semantic system. Biling. Lang. Cogn..

[40] Gollan TH, Montoya RI, Fennema-Notestine C, Morris SK (2005). Bilingualism affects picture naming but not picture classification. Mem. Cogn..

[41] Gollan TH, Montoya RI, Cera C, Sandoval TC (2008). More use almost always means a smaller frequency effect: Aging, bilingualism, and the weaker links hypothesis. J. Mem. Lang..

[42] Bialystok E (2017). The Bilingual Adaptation: How Minds Accommodate Experience. Psychol. Bull..

[43] Paap KR, Johnson HA, Sawi O (2015). Bilingual advantages in executive functioning either do not exist or are restricted to very specific and undetermined circumstances. Cortex.

[44] Samuel S, Roehr-Brackin K, Pak H, Kim H (2018). Cultural Effects Rather Than a Bilingual Advantage in Cognition: A Review and an Empirical Study. Cogn. Sci..

[45] Dick AS (2019). No bilingual advantage for executive function: Evidence from a large sample of children in the Adolescent Brain and Cognitive Development (ABCD) Study. Nature Human Behavior.

[46] Antón E, Carreiras M, Andoni JA (2019). The impact of bilingualism on executive functions and working memory in young adults. PLoS One.

[47] Lehtonen, M. *et al*. Is Bilingualism Associated with Enhanced Executive Functioning in Adults? A Meta-Analytic Review. *Psychol. Bull*. **144** (2018). 10.1037/bul0000142 29494195

[48] Wu J, Chen Y, van Heuven VJ, Schiller NO (2018). Dynamic effect of tonal similarity in bilingual auditory lexical processing. Lang. Cogn. Neurosci..

[49] Neergaard Karl D., Huang Chu-Ren (2019). Constructing the Mandarin Phonological Network: Novel Syllable Inventory Used to Identify Schematic Segmentation. Complexity.

[50] Olguin A, Cekic M, Bekinschtein TA, Katsos N, Bozic M (2019). Bilingualism and language similarity modify the neural mechanisms of selective attention. Sci. Rep..

[51] Chéreau C, Gaskell MG, Dumay N (2007). Reading spoken words: Orthographic effects in auditory priming. Cognition.

[52] Pattamadilok C, Perre L, Dufau S, Ziegler JC (2009). On-line Orthographic Influences on Spoken Language in a Semantic Task. J. Cogn. Neurosci..

[53] Ziegler JC, Muneaux M, Grainger J (2003). Neighborhood effects in auditory word recognition: Phonological competition and orthographic facilitation. J. Mem. Lang..

[54] Muneaux M, Ziegler JC (2004). Locus of orthographic effects in spoken word recognition: Novel insights from the neighbour generation task. Lang. Cogn. Process..

[55] Montant M, Schön D, Anton J, Ziegler JC (2011). Orthographic contamination of Broca’s area. Front. Psychol..

[56] Perre L, Pattamadilok C, Montant M, Ziegler JC (2009). Orthographic effects in spoken language: On-line activation or phonological restructuring?. Brain Res..

[57] Cao, F. & Perfetti, C. A. Neural signatures of the reading-writing connection: Greater involvement of writing in Chinese reading than english reading. *PLoS One* **11** (2016). 10.1371/journal.pone.0168414 PMC5161366 27992505

[58] Brennan C, Cao F, Pedroarena-Leal N, McNorgan C, Booth JR (2013). Reading acquisition reorganizes the phonological awareness network only in alphabetic writing systems. Hum. Brain Mapp..

[59] Cao F, Brennan C, Booth JR (2015). The brain adapts to orthography with experience: Evidence from English and Chinese. Dev. Sci..

[60] Cao F (2011). Development of brain networks involved in spoken word processing of Mandarin Chinese. Neuroimage.

[61] Rastle K, McCormick SF, Bayliss L, Davis CJ (2011). Orthography Influences the Perception and Production of Speech. J. Exp. Psychol. Learn. Mem. Cogn..

[62] Bürki A, Spinelli E, Gaskell MG (2012). A written word is worth a thousand spoken words: The influence of spelling on spoken-word production. J. Mem. Lang..

[63] Qu Qingqing, Damian Markus F (2019). The role of orthography in second-language spoken word production: Evidence from Tibetan Chinese bilinguals. Quarterly Journal of Experimental Psychology.

[64] Qu Q, Damian MF (2019). Orthographic effects in Mandarin spoken language production. Mem. Cognit..

[65] Verdonschot, R. G., Nakayama, M., Zhang, Q., Tamaoka, K. & Schiller, N. O. The Proximate Phonological Unit of Chinese-English Bilinguals: Proficiency Matters. *PLoS One* **8** (2013). 10.1371/journal.pone.0061454 PMC3640013 23646107

[66] Li C, Wang M, Davis JA (2015). The phonological preparation unit in spoken word production in a second language. Biling. Lang. Cogn..

[67] Zhou, Y. The Historical Evolution of Chinese Languages and Scripts. Pathways to Advanced Skills Series, Volume 8. (Ohio State University National East Asian Languages Resource Center, 2003).

[68] Siew, C. S. Q., Wulff, D. U., Beckage, N. M. & Kenett, Y. N. Cognitive Network Science: A Review of Research on Cognition through the Lens of Network Representations, Processes, and Dynamics. *Complexity* 1–24, 10.1155/2019/2108423%0AReview (2019).

[69] Dubossarsky H, De Deyne S, Hills TT (2017). Quantifying the Structure of Free Association Networks Across the Life Span. Dev. Psychol..

[70] Vitevitch MS, Goldstein R (2014). Keywords in the mental lexicon. J. Mem. Lang..

[71] Siew CSQ (2018). The orthographic similarity structure of English words: Insights from network science. Appl. Netw. Sci..

[72] Siew CSQ, Vitevitch MS (2019). The phonographic language network: Using network science to investigate the phonological and orthographic similarity structure of language. J. Exp. Psychol. Gen..

[73] Stella, M., Beckage, N. M., Brede, M. & De Domenico, M. Multiplex model of mental lexicon reveals explosive learning in humans. *Sci. Rep*. **8** (2018). 10.1038/s41598-018-20730-5 PMC5797130 29396497

[74] Stella, M. & Brede, M. Patterns in the English Language: Phonological Networks, Percolation and Assembly Models. *J. Stat. Mech. Theory Exp*. **5** (2015).

[75] Borodkin K, Kenett YN, Faust M, Mashal N (2016). When pumpkin is closer to onion than to squash: The structure of the second language lexicon. Cognition.

[76] Kenett YN (2013). Semantic organization in children with cochlear implants: computational analysis of verbal fluency. Front. Psychol..

[77] Bertola L (2014). Graph analysis of verbal fluency test discriminate between patients with Alzheimer’s disease, mild cognitive impairment and normal elderly controls. Front. Aging Neurosci..

[78] Zemla JC, Austerweil JL (2018). Estimating Semantic Networks of Groups and Individuals from Fluency Data. Comput. Brain Behav..

[79] Goñi J (2012). The semantic organization of the animal category: evidence from semantic verbal fluency and network theory. Cogn. Process..

[80] Levelt, W. J. M. Speaking: From intention to articulation. (MIT Press, 1989).

[81] Vitevitch MS (2008). What can graph theory tell us about word learning and lexical retrieval?. J. Speech, Lang. Hear. Res..

[82] Turnbull, R. & Peperkamp, S. What governs a language’s lexicon? Determining the organizing principles of phonological neighbourhood networks. In Proceedings of the 5th International Workshop on Complex Networks and their Applications (COMPLEX NETWORKS 2016) (eds Cherifi, H., Gaito, S., Quattrociocchi, W. & Sala, A.) 83–94 (Springer, Cham, 2016).

[83] Arbesman S, Strogatz SH, Vitevitch MS (2010). The Structure of Phonological Networks Across Multiple Languages. Int. J. Bifurc. Chaos.

[84] Arbesman S, Strogatz SH, Vitevitch MS (2010). Comparative analysis of networks of phonologically similar words in English and Spanish. Entropy.

[85] Brown KS (2018). Universal Features in Phonological Neighbor Networks. Entropy.

[86] Shoemark, P., Goldwater, S., Kirby, J. & Sarkar, R. Towards robust cross-linguistic comparisons of phonological networks Towards robust cross-linguistic comparisons of phonological networks. in the 14th Annual SIGMORPHON Workshop on Computational Research in Phonetics, *Phonology, and Morphology* 110–120, 10.18653/v1/W16-2018 (2016).

[87] Siew CSQ (2013). Community structure in the phonological network. Front. Psychol..

[88] Siew CSQ, Vitevitch MS (2015). Spoken word recognition and serial recall of words from componenets in the phonological network. J. Exp. Psychol. Learn. Mem. Cogn. Cogn.

[89] Chan KY, Vitevitch MS (2010). Network Structure Influences Speech Production. Cogn. Sci..

[90] Chan KY, Vitevitch MS (2009). The influence of the phonological neighborhood clustering coefficient on spoken word recognition. J. Exp. Psychol. Hum. Percept. Perform..

[91] Vitevitch MS, Ercal G, Adagarla B (2011). Simulating retrieval from a highly clustered network: implications for spoken word recognition. Front. Psychol..

[92] Burt, C. The Distribution and Relations of Educational Abilities. (London County Council, 1917).

[93] Thurstone, L. L. Primary Mental Abilities. (University of Chicago Press, 1938).

[94] Troyer AK, Moscovitch M, Winocur G (1997). Clustering and switching as two components of verbal fluency: Evidence from younger and older healthy adults. Neuropsychology.

[95] Luce PA, Pisoni DB (1998). Recognizing Spoken Words: The Neighborhood Activation Model. Ear Hear..

[96] Woodcock, R. W., Alvarado, C. G. & Ruef, M. L. Woodcock-Muñoz Language Survey III. (Houghton, Mifflin Harcourt, 2017).

[97] Marian V, Blumenfeld HK, Kaushanskaya M (2007). The Language Experience and Proficiency Questionnaire (LEAP-Q): Assessing Language Profiles in Bilinguals and Multilinguals. J. Speech, Lang. Hear. Res..

[98] Li P, Sepanski S, Zhao X (2006). Language history questionnaire: A Web-based interface for bilingual research. Behav. Res. Methods.

[99] Ma W, Winke P (2019). Self-assessment: How reliable is it in assessing oral proficiency over time?. Foreign Lang. Ann..

[100] Tigchelaar, M. Exploring the Relationship Between Self- Assessments and OPIc Ratings of Oral Proficiency in French. in Foreign language proficiency in higher education (eds Winke, P. & Gass, S. M.) 153–173 (Springer, 2019).

[101] Malabonga V, Kenyon DM, Carpenter H (2005). Self-assessment, preparation and response time on a computerized oral proficiency test. Langauge Test..

[102] Neergaard, K. D., Xu, H. & Huang, C.-R. Database of Mandarin neighborhood statistics. in Proceedings of the 10th International Conference on Language Resources and Evaluation, LREC 2016 (2016).

[103] Fisk J, Sharp CA (2004). Age-Related Impairment in Executive Functioning: Updating, Inhibition, Shifting, and Access. J. Clin. Exp. Neuropsychol..

[104] Wiener S, Turnbull R (2015). Constraints of Tones, Vowels and Consonants on Lexical Selection in Mandarin Chinese. Lang. Speech.

[105] Csárdi G, Nepusz T (2006). The igraph software package for complex network research. InterJournal Complex Syst..

[106] Castro N, Pelczarski KM, Vitevitch MS (2017). Using Network Science Measures to Predict the Lexical Decision Performance of Adults Who Stutter. J. Speech Lang. Hear. Res..

[107] Goldstein R, Vitevitch MS (2014). The influence of clustering coefficient on word-learning: how groups of similar sounding words facilitate acquisition. Front. Psychol..

[108] Bonat, W. H. Multiple Response Variables Regression Models in R: The mcglm Package. *J. Stat. Softw*. **84** (2018).

[109] Bonat WH, Kokonendji CC (2016). Flexible Tweedie regression models for continuous data. J. Stat. Comput. Simul..

[110] Bonat WH, Jørgensen B, Kokonendji CC, Hinde J, Demétrio CGB (2017). Extended Poisson–Tweedie: Properties and regression models for count data. Stat. Modelling.

[111] Wood SN, Scheipl F, Faraway JJ (2013). Straightforward intermediate rank tensor product smoothing in mixed models. Stat. Comput..

[112] Newman MEJ (2002). Assortative Mixing in Networks. Phys. Rev. Lett..

[113] Vitevitch MS, Chan KY, Goldstein R (2014). Insights into failed lexical retrieval from network science. Cogn. Psychol..

[114] Strauss TJ, Harris HD, Magnuson JS (2007). jTRACE: A reimplementation and extension of the TRACE model of speech perception and spoken word recognition. Behav. Res. Methods.

[115] Wulff, D. U., Hills, T. T. & Mata, R. Structural differences in the semantic networks of younger and older adults. PsyArXiv October, (2018). 10.1038/s41598-022-11698-4 PMC9744829 36509768

[116] Vitevitch MS, Luce PA (1998). When words complete: Levels of processing in perception of spoken words. Psychol. Sci..

[117] Neergaard, K. D., Britton, J. & Huang, C.-R. Neighborhood in Decay: Working Memory Modulates Effect of Phonological Similarity on Lexical Access. in Proceedings of the 41st Annual Conference of the Cognitive Science Society (eds Goel, A. K., Seifert, C. M. & Freksa, C.) 2447–2453 (Cognitive Science Society, 2019).

[118] Vitevitch MS, Rodríguez E (2004). Neighborhood density effects in spoken word recognition in Spanish. J. Multiling. Commun. Disord..

[119] Sanchez SV (2013). A Case for Multidimensional Bilingual Assessment. Lang. Assess. Q..

