# Author Correction: Phonological network fluency identifies phonological restructuring through mental search

**DOI:** 10.1038/s41598-019-55966-2

**Published:** 2020-01-14

**Authors:** Karl David Neergaard, Jin Luo, Chu-Ren Huang

**Affiliations:** 1 University of Macau, Department of English, Macau, S.A.R. China; 2 Aix-Marseille University, Laboratoire Parole et Langage, Aix-en-Provence, 13100 France; 3 University of Groningen, Erasmus+ Mundus Joint Master Degree in Clinical Linguistics, 9712 Groningen, The Netherlands; 4 The Hong Kong Polytechnic University, Department of Chinese and Bilingual Studies, Hong Kong, S.A.R. China

Correction to: *Scientific Reports* 10.1038/s41598-019-52433-w, published online 05 November 2019

The HTML and PDF versions of this Article contained a typographical error in the spelling of the author Chu-Ren Huang, which was incorrectly given as Chu -Ren Huang.

In addition, the original version of this Article contained errors in the Abstract.

“Post exclusion, 95 native-language Mandarin speakers produced as many items that differed by a single lexical tone as possible within one minute.”.

now reads:

“Post exclusion, 95 native-language Mandarin speakers produced as many items that differed by a single segment or lexical tone as possible within one minute.”.

Lastly, the original article contains errors in the Methods section under the subheading ‘Fluency’.

“We introduce ‘weighted edit’ (WE), shown in Fig. 1b. WE states that a given word’s value to the final fluency score decreases as edit distance between itself and the auditory stimuli increases. Fluency weightings differed according to the number of units for each of the stimuli and production, such that weightings were the proportion of the number of edit distances possible prior to two items sharing no similarity. For instance, for four-unit stimuli that received five-unit responses, edit 1 garnered 1 point (e.g., wai4/uaɪ^5151^/→ shuai4/ʂuaɪ^5151^/), edit 2 = 0.75 (e.g., wai4/uaɪ^5151^/→ shuan4/ ʂuan^5151^/), edit 3 = 0.50 (e.g., wai4/uaɪ^5151^/→ xiang4/ɕiaŋ^5151^/), edit 4 = 0.25 (e.g., wai4/uai^5151^/→ xiang3/ɕiaŋ^214^/), and edit 5 = 0 (e.g., wai4/uaɪ^5151^/→ xiong3/ɕioŋ^214^/). Similarly, for three-unit stimuli that received two-unit responses, edit 1 = 1 (e.g., du2/tu^3535^/→ wu2/u^3535^/), edit 2 = 0.50 (e.g., tu2/tu^3535^/→ wu4/u^5151^/), edit 3 = 0 (e.g., tu2/tu^3535^/→ yi4/i^5151^/), edits 4 and 5 also received 0 points due to not being possible combinations.”

now reads:

“We introduce ‘weighted edit’ (WE), shown in Fig. 1b. WE states that a given word’s value to the final fluency score decreases as edit distance between itself and the auditory stimuli increases. Fluency weightings differed according to the number of units for each of the stimuli and production, such that weightings were the proportion of the number of edit distances possible prior to two items sharing no similarity. For instance, for four-unit stimuli that received five-unit responses, edit 1 garnered 1 point (e.g., wai4/uaɪ^51^/→ shuai4/ʂuaɪ^51^/), edit 2 = 0.75 (e.g., wai4/uaɪ^51^/→ shuan4/ʂuan^51^/), edit 3 = 0.50 (e.g., wai4/uaɪ^51^/→ xiang4/ɕiaŋ^51^/), edit 4 = 0.25 (e.g., wai4/uaɪ^51^/→ xiang3/ɕiaŋ^214^/), and edit 5 = 0 (e.g., wai4/uaɪ^51^/→ xiong3/ɕioŋ^214^/). Similarly, for three-unit stimuli that received two-unit responses, edit 1 = 1 (e.g., du2/tu^35^/→ wu2/u^35^/), edit 2 = 0.50 (e.g., tu2/tu^35^/→ wu4/u^51^/), edit 3 = 0 (e.g., tu2/tu^35^/→ yi4/i^51^/), edits 4 and 5 also received 0 points due to not being possible combinations.”

These errors have now been corrected in the PDF and HTML versions of the published Article.

